# Developmental Impairments in a Rat Model of Methyl Donor Deficiency: Effects of a Late Maternal Supplementation with Folic Acid

**DOI:** 10.3390/ijms20040973

**Published:** 2019-02-23

**Authors:** Andréa Geoffroy, Lynda Saber-Cherif, Grégory Pourié, Déborah Helle, Rémy Umoret, Jean-Louis Guéant, Carine Bossenmeyer-Pourié, Jean-Luc Daval

**Affiliations:** Université de Lorraine, Inserm U1256, NGERE, Faculté de Médecine, 9 avenue de la Forêt de Haye, F-54500 Vandoeuvre-lès-Nancy, France; g.andrea@hotmail.fr (A.G.); lynda.saber-cherif@univ-lorraine.fr (L.S.-C.); gregory.pourie@univ-lorraine.fr (G.P.); deborah.helle@univ-lorraine.fr (D.H.); remy.umoret@univ-lorraine.fr (R.U.); jean-louis.gueant@univ-lorraine.fr (J.-L.G.)

**Keywords:** development, postnatal brain maturation, folate, vitamin B12, microRNAs, maternal folate supplementation during lactation

## Abstract

Vitamins B9 (folate) and B12 act as methyl donors in the one-carbon metabolism which influences epigenetic mechanisms. We previously showed that an embryofetal deficiency of vitamins B9 and B12 in the rat increased brain expression of let-7a and miR-34a microRNAs involved in the developmental control of gene expression. This was reversed by the maternal supply with folic acid (3 mg/kg/day) during the last third of gestation, resulting in a significant reduction of associated birth defects. Since the postnatal brain is subject to intensive developmental processes, we tested whether further folate supplementation during lactation could bring additional benefits. Vitamin deficiency resulted in weaned pups (21 days) in growth retardation, delayed ossification, brain atrophy and cognitive deficits, along with unchanged brain level of let-7a and decreased expression of miR-34a and miR-23a. Whereas maternal folic acid supplementation helped restore the levels of affected microRNAs, it led to a reduction of structural and functional defects taking place during the perinatal/postnatal periods, such as learning/memory capacities. Our data suggest that a gestational B-vitamin deficiency could affect the temporal control of the microRNA regulation required for normal development. Moreover, they also point out that the continuation of folate supplementation after birth may help to ameliorate neurological symptoms commonly associated with developmental deficiencies in folate and B12.

## 1. Introduction

Numerous studies have shown that epigenetic regulations such as DNA methylation and histone modification play a critical role in genomic programming during development, by regulating cell proliferation and differentiation [1,2,3]. In this respect, the methyl donors folate (vitamin B9) and vitamin B12 are cofactors in the one-carbon metabolism that plays a key role in transmethylation reactions [4,5]. They promote the remethylation of homocysteine to methionine and are essential for the synthesis of S-adenosylmethionine (SAM), the universal methyl donor for biological methylations [6]. In addition, folate and B12 are key regulators of the concentration of homocysteine which can exert adverse effects such as DNA breakage, oxidative stress, endoplasmic reticulum stress, protein homocysteinylation, and apoptosis [7,8,9,10]. Both vitamins are essential for normal development, and their insufficiency constitutes a risk factor for various developmental disorders such as congenital heart defects, Down syndrome, and particularly neural tube defects [11,12,13]. This led to public health policies recommending periconceptional supplementation with folic acid, usually until the end of the first trimester of pregnancy [14,15,16,17,18]. However, if prenatal life encompasses foundational and critical phases, brain development and maturation continue during the perinatal and postnatal periods, and it has been largely documented that even subtle alterations of the prenatal environment may have meaningful consequences for later development and functional maturation [19,20,21].

In previous studies, we showed that a methyl donor deficiency (folate and B12) during the embryofetal period in the rat affects the brain expression of several microRNAs that participate in the developmental control of gene expression, impacting their downstream signaling pathways [22,23]. This process could be reversed by a maternal supply with folic acid during the last third of gestation, resulting in a significant mitigation of methyl donor deficiency-associated defects at birth [23].

By using the same experimental model, we therefore aimed to evaluate whether continued folate supplementation during the lactating period until weaning (postnatal day 21 in rats) could bring additional benefits, notably on brain functional maturation. Taken together, our data showed that in utero deficiency in folate and vitamin B12 was associated postnatally with persisting growth retardation and morphological and functional defects, in line with misexpression of miR-34a and miR-23a. Importantly, perinatal and postnatal exposure to supplementation with folic acid restored microRNA levels and proved to be effective in reducing deficiency-related alterations.

## 2. Results

### 2.1. Folate, Vitamin B12, and Homocysteine Plasma Concentrations

**Figure 1:**
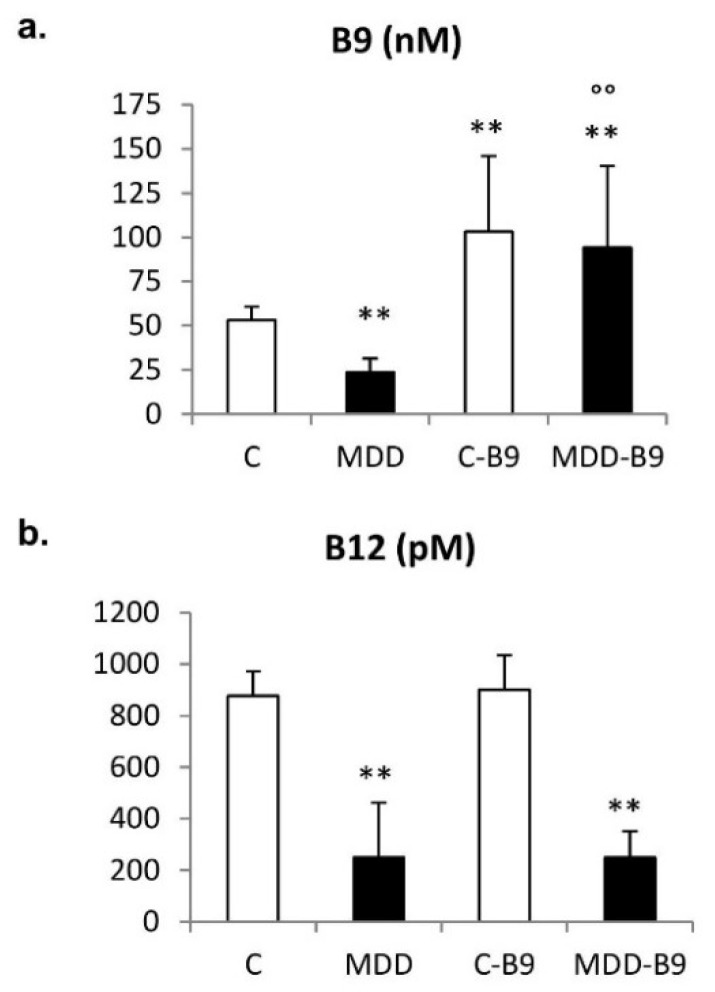

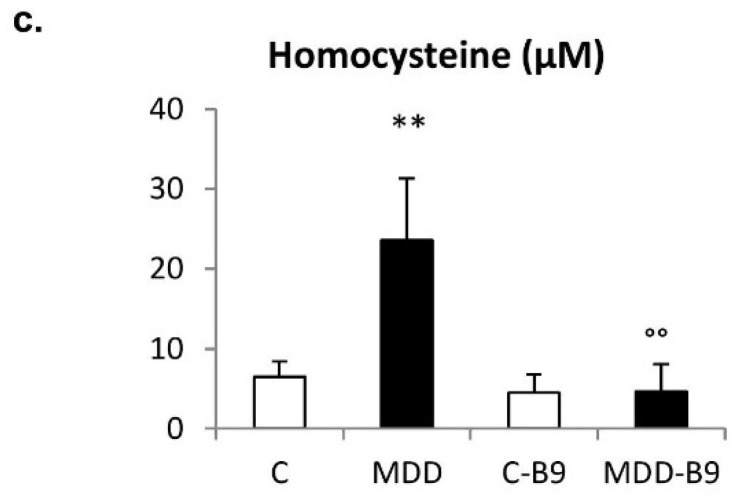
Effects of the maternal dietary regimen on plasma concentrations of folate (**a**), vitamin B12 (**b**) and homocysteine (**c**) in 21-day-old rat pups. Data are mean ± SD and were obtained from 14 ≤ *n* ≤39 individuals. Statistically significant differences: ** *p* < 0.01: With respective control; °° *p* < 0.01: Between MDD and MDD + B9. C = control diet; MDD = methyl donor deficient diet.

As a consequence of the maternal deficiency, plasma levels of folate and vitamin B12 were dramatically reduced in the rat progeny at weaning (postnatal day 21). Concomitantly, homocysteinemia was significantly augmented (*p* < 0.01). Figure 1 shows that folic acid supplementation restored folate concentration without affecting the vitamin B12 status, and significantly reduced hyperhomocysteinemia in deficient pups.

### 2.2. Growth Retardation and Developmental Abnormalities

**Figure 2:**
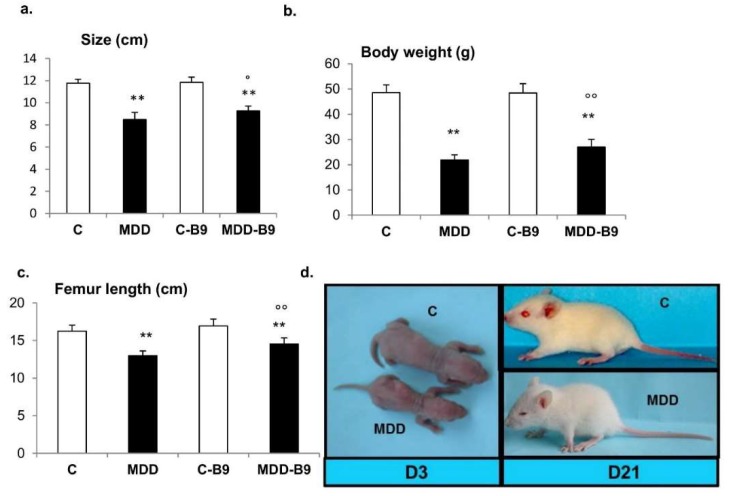
Effects of maternal methyl donor deficiency and folic acid supplementation on rat pup morphometric properties. (**a**–**c**) General morphometric measurements in control (C), methyl donor deficient (MDD), supplemented control (C-B9) and supplemented deficient (MDD-B9) rat pups at 21 days of age (20 ≤ *n* ≤ 40). Data are reported as mean ± standard deviation. Statistically significant differences between control and MDD rats: ** *p* < 0.01, between MDD and supplemented MDD: ° *p* < 0.05 and °° *p* < 0.01; (**d**) Illustration of MDD-associated growth retardation at 3 and 21 postnatal days.

In rat pups born to deficient dams, the body length was reduced by 26% as compared to controls at 21 days of age, whereas the body weight was decreased by 55% and femur length, reflecting pre- and postnatal growth, was reduced by 21% (Figure 2). The stunting rate could be ameliorated by a maternal supplementation with folic acid (Figure 2).

**Figure 3:**
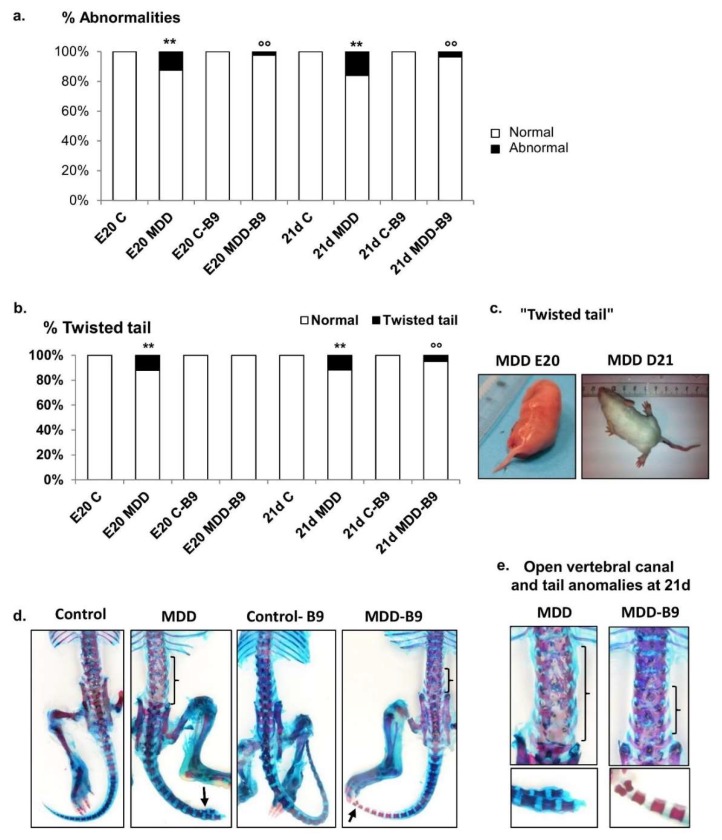
Effects of maternal methyl donor deficiency and folic acid supplementation on developmental abnormalities in the progeny. (**a**) Occurrence of developmental abnormalities in control and MDD fetuses at embryonic day 20 (E20) and at postnatal day 21 (21 d), and following folic acid (B9) supplementation (20 ≤ *n* ≤ 40). Statistically significant differences between control and MDD rats: ** *p* < 0.01, between MDD and supplemented MDD: °° *p* < 0.01; (**b**) Prevalence of “twisted tail” in controls, MMD and supplemented rats; (**c**) Illustration of MDD-associated “twisted tail” at E20 and postnatal day 21; (**d**,**e**) Photographs of the vertebral canal and tail (Alcian blue/Alizarin red staining) in the various experimental groups at 21 days (square brackets delineate open canal).

By contrast to controls, 12.5% of deficient fetuses (E20) and 15% of deficient pups at postnatal day 21 were affected by at least one morphological anomaly such as syndactyly, atrophied digits, as well as signs indicative of spina bifida, such as “twisted tail” and open vertebral canal, in addition to delayed ossification and fused vertebrae in the lumbosacral region, as illustrated in Figure 3. Importantly, the occurrence of abnormalities was consistently reduced following folic acid supplementation that improved cartilage ossification, ameliorated spinal canal phenotype and tail morphology in deficient offspring.

**Figure 4:**
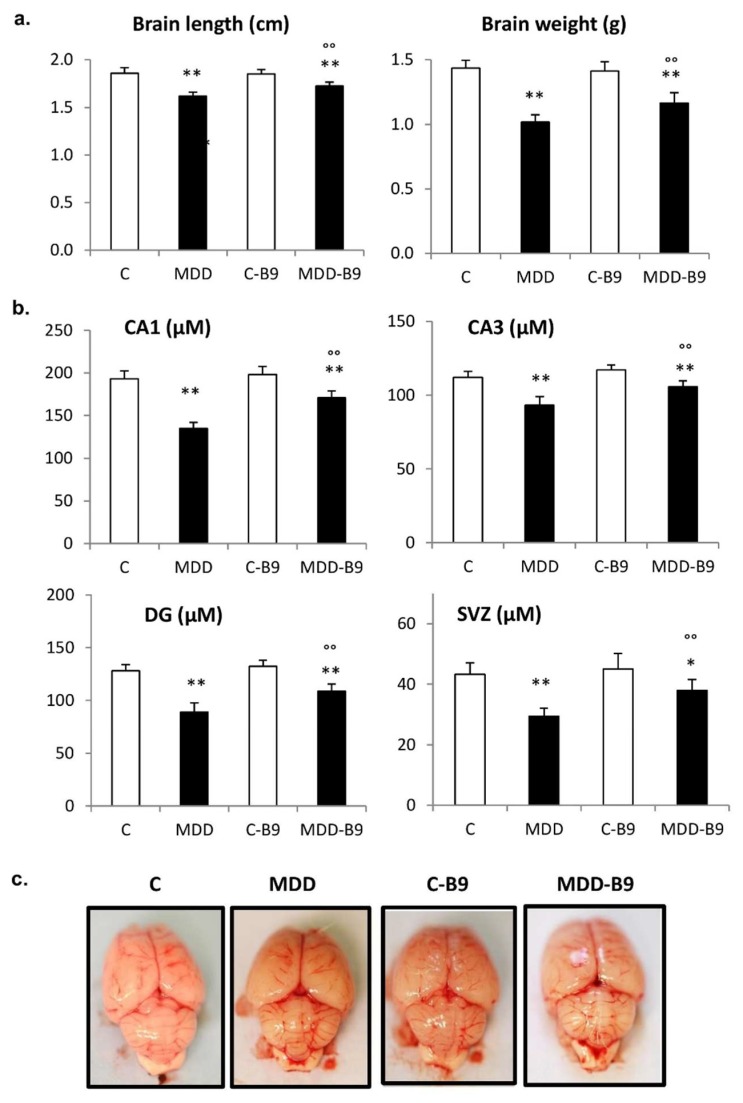
Brain defects associated with maternal methyl donor deficiency in rat pups: influence of folic acid supplementation. (**a**) Brain length and weight in control (C), methyl donor deficient (MDD), supplemented control (C-B9) and supplemented deficient (MDD-B9) rat pups at 21 days (20 ≤ *n* ≤ 40). Statistically significant differences between control and MDD rats: ** *p* < 0.01, between MDD and supplemented MDD: °° *p* < 0.01; (**b**) thicknesses of hippocampal CA1 pyramidal cell layer, CA3 pyramidal cell layer, granular cell layer of the dentate gyrus (DG), and subventricular zone (SVZ) at 21 days (6 ≤ *n* ≤ 10). Statistically significant differences between control and MDD rats: * *p* < 0.05 and ** *p* < 0.01, between MDD and supplemented MDD: °° *p* < 0.01; (**c**) Photographs of representative brain samples showing the absence of macroscopic defects (e.g., improper interhemispheric junction and cerebellar vermis closure) at 21 days regardless of the experimental group.

The brain size and weight were significantly reduced following exposure to methyl donor deficiency (by 12% and 29%, respectively) (Figure 4). In the hippocampus, the thicknesses of CA1 and CA3 pyramidal cell layers as well as that of the granular cell layer of the dentate gyrus were dramatically affected (about 30%). The same observation was made for the neurogenic subventricular zone (SVZ). In all cases, maternal supplementation with folic acid allowed significant reduction of these defects.

In a previous study using the same experimental conditions [23], we reported that gestational methyl donor deficiency was associated, at the embryonic stage E20, with the delayed closure of the cephalic parts of the neural tube, as reflected by an improper interhemispheric junction and open cerebellar vermis. The occurrence of open cerebellar vermis at E20 varied from 41% in deficient fetuses to ~3% in controls. By contrast, as illustrated in Figure 4c, no default could be observed at postnatal day 21, regardless of the experimental group.

### 2.3. Expression of let-7a, miR-34a and miR-23a

**Figure 5:**
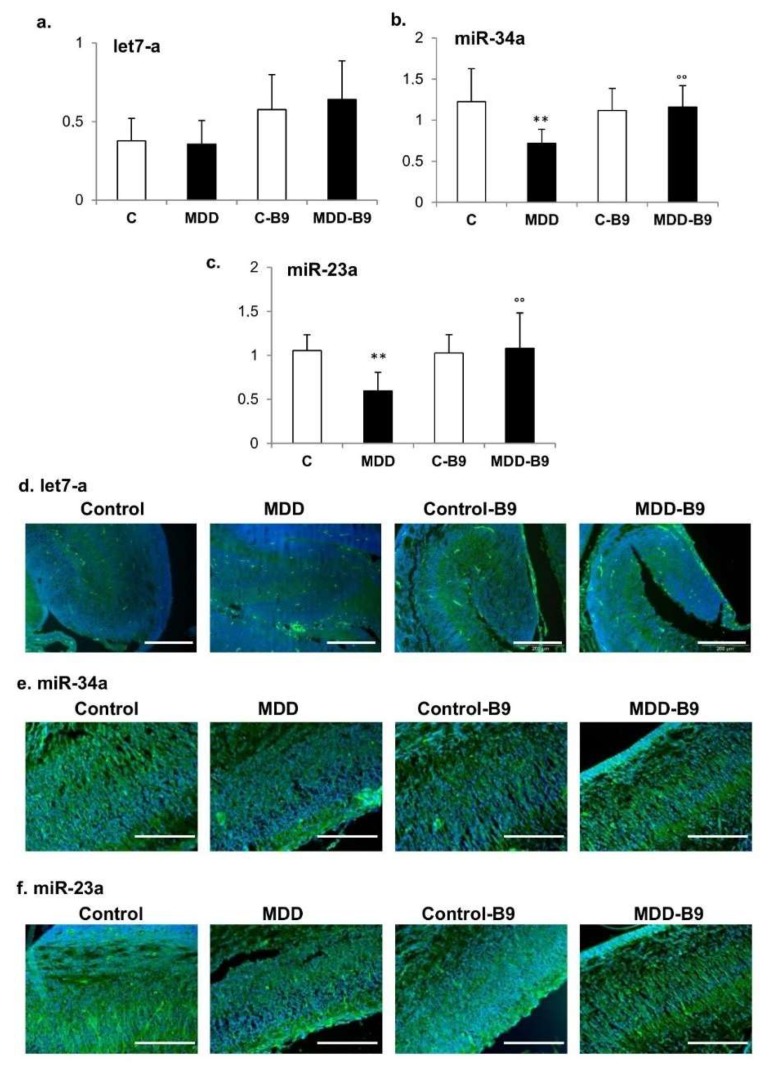
Effects of maternal methyl donor deficiency on the expression of let-7a, miR-34a and miR-23: influence of folic acid supplementation. (**a**–**c**) Expression levels of let-7a (**a**), miR-34 (**b**) and miR-23 (**c**) in arbitrary units in the hippocampus of control (C) and deficient (MDD) rat pups at 21 days, and effects of folic acid (B9) supplementation. Data are reported as mean ± SD (6 ≤ *n* ≤ 8). Statistically significant differences between control and MDD: ** *p* < 0.01, between MDD and supplemented MDD: °° *p* < 0.01; (**d**–**f**) Effects of methyl donor deficiency and folic acid supplementation on the expression of Let-7a (**d**), miR-34a (**e**) and miR-23 (**f**) as depicted by in situ hybridization in the hippocampus from rat pups at 21 days. The scale bar corresponds to 200 µm.

When studied by TaqMan RT-qPCR, the let-7a expression level remained unchanged in extracts of hippocampal tissues from deficient pups (Figure 5a), whereas miR-34a level was significantly decreased (42%), an effect that was reversed by folic acid supplementation (Figure 5b). Similar observations could be made regarding the expression of miR-23a, which was reduced in the same proportions as miR-34 under deficiency conditions (Figure 5c). The data were confirmed by in situ hybridization in hippocampus tissue sections (Figure 5d–f).

### 2.4. Neurobehavioral Development

**Figure 6:**
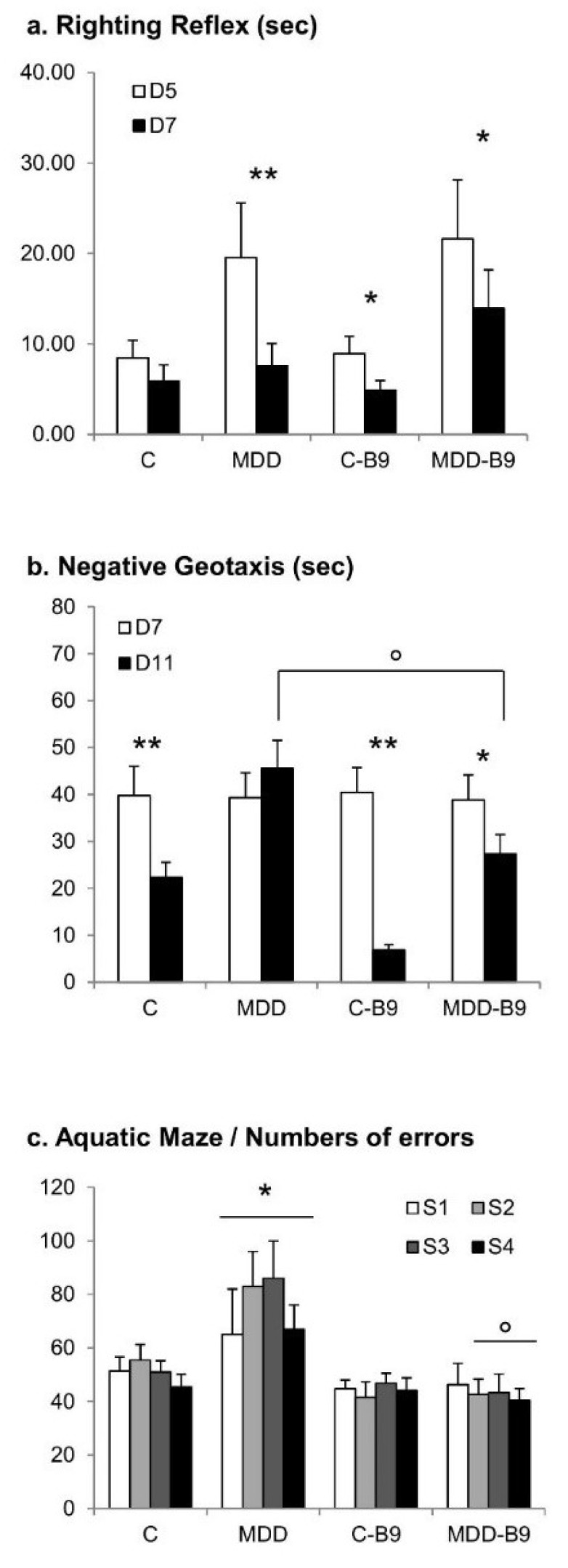
Effects of maternal methyl donor deficiency and folic acid supplementation on rat pup neurobehavioral development. (**a**) Sensorimotor righting reflex monitored at 5 and 7 days of age; (**b**) Negative geotaxis monitored daily between 7 and 11 days of age; (**c**) Learning and memory capacities capacities as reflected by the number of errors in the water-maze from 21 to 24 days of age (sessions 1–4). Data are reported as mean ± SD (9 ≤ *n* ≤ 26). Statistically significant differences between control and MDD: * *p* < 0.05 and ** *p* < 0.01, between MDD and supplemented MDD: ° *p* < 0.05.

Neurobehavioral development was evaluated through three tests, i.e., the righting reflex at postnatal days 5 and 7, the negative geotaxis at postnatal days 7–11 (both monitoring sensorimotor scores), and the aquatic maze at postnatal days 21–24 (evaluating learning performances). Regarding the early righting reflex, the time necessary to come back to a quadruped position was transiently but significantly increased in the deficient group compared to the control group on the first day of testing, whereas folate supplementation had no significant effect (Figure 6a). In the negative geotaxis test, the time needed to turn up completely in the slope decreased significantly between 7 days and 11 days of age in control pups, indicating improved scores with time. Such amelioration was not observed in deficient pups. Furthermore, folic acid exerted beneficial effects, with improved scores recorded at day 11 compared to day 7 (Figure 6b). Finally, the monitoring of hippocampus-dependent cognitive performances showed that deficient pups always displayed a higher number of errors than controls, reflecting poorer abilities to learn how to escape the maze (Figure 6c). It is noticeable that maternal supplementation improved the learning scores of deficient pups.

## 3. Discussion

In our animal model, maternal exposure to methyl donor deficiency is associated with a global growth retardation and brain atrophy, particularly affecting hippocampal cell layers and the neurogenic subventricular zone. We have previously reported various developmental abnormalities, such as spontaneous abortion, congenital malformations, delayed ossification, and a high prevalence of neural tube defects [22,23]. Anomalies previously observed at birth (embryonic day 20) are still present at postnatal day 21. However, whereas we frequently observed improper interhemispheric junction and unclosed cerebellar vermis at E20 [23], none of these defaults could be detected at 21 days, suggesting they are transient and reflect a developmental delay. Nevertheless, sensorimotor properties were shown to be affected in methyl donor deficient pups, while deficits in learning and memory retrieval capacities were recorded at 21–24 days of age, in good accordance with morphologic alterations of the hippocampus.

Folate deficiency is well known to affect fetal and neonatal brain development and can result in various functional deficits [12,24], and the resulting developmental disorders are mediated by a wide range of underlying mechanisms. These include the participation of microRNAs, small noncoding RNAs that regulate target gene expression, which in turn influences cell cycling, cell differentiation, and apoptosis during development [25,26,27,28]. In this respect, we have previously shown that gestational deficit in folate and B12 led to a significantly increased brain expression of let-7a and miR-34a in rat fetuses at E20, with subsequent downregulation of their regulatory targets, i.e., Trim71 and Notch signaling partners, respectively [23]. Let-7-associated pathways have been described as key regulators of neural cell proliferation and differentiation [29], and has been tightly associated with the occurrence of neural tube defects [30]. This is consistent with the relatively high prevalence of anomalies of the cephalic part of the neural tube previously depicted in E20 rat pups [23]. miR-34a regulates numerous target genes involved in cell cycle, apoptosis, differentiation, and neuron maintenance [31]. The associated Notch signaling pathway plays multiple roles in the development of the central nervous system including regulating neural stem cell proliferation, survival, self-renewal and differentiation [32]. Interestingly, in the present study, we observed no significant change in the hippocampal expression of let-7a and a decreased expression of miR-34a in deficient pups at 21 days of age. This contrasts with our previous data obtained at E20. Since normal development requires a series of programming processes involving accurate time-controlled gene activation/silencing expressions, our results suggest a desynchronization of the developmental program in conditions of methyl donor deficiency. In other words, gestational vitamin deficiency could modify the windows of normal expression of various microRNAs, affecting their related pathways involved in proper development and influencing the proliferation/differentiation balance.

In addition, we also showed a repression of miR-23a in the brain of 21-day-old rat pups following maternal deficiency. First reported as astrocyte specific [33], miR-23 was shown to be strongly expressed in neuroepithelium progenitor cells in the developing spinal cord [34]. It may play a role in cell proliferation [35] and apoptosis [36,37], and has been described as a key actor for myelination [38,39]. It was also suggested that miR-23a is involved in osteogenic differentiation, as shown in in vitro studies [40].

Although not investigated in the present study because it is less involved in methylation processes, vitamin B6 (pyridoxine) is a cofactor of cystathionine beta-synthase that contributes to homocysteine metabolism through the transsulfuration pathway [6]. When it occurs, particularly in the elderly, a vitamin B6 deficiency inhibits the conversion of homocysteine to cystathionine and thus may also cause hyperhomocysteinemia [41].

Early gestational administration of folic acid is recognized to exert beneficial effects, especially for the prevention of neural tube defects such as spina bifida [16,17]. We recently reported that folate supplementation during the third week of gestation in the rat (corresponding to the last trimester in humans) alleviates methyl donor deficiency-associated birth defects [23]. However, the effects of further processing during the postnatal period corresponding to a peak of brain maturation were not documented. Indeed, numerous neuronal adaptative changes occur during this period, and classic developmental events of neural maturation, circuit refinement and plasticity are known to be influenced significantly by both spontaneous and experience-dependent activities [42,43,44]. As for gestational supplementation, the maternal administration of folic acid during lactation reduced the frequently observed developmental anomalies following B vitamin deficiency such as growth retardation, delayed ossification, and even signs of neural tube alteration, in line with the restoration of affected microRNAs such as miR-34a and miR-23a, as compared to untreated animals. Most importantly, brain atrophy and functional deficits could be reduced by late folate supply. This suggests that the continuation of folate supplementation after birth may help to ameliorate cognitive functions as those described lately after in utero deficit in folate/vitamin B12 [12,45], as outlined by Hochberg et al. [46] in their review where they address the influence of nutrition in developmental plasticity and epigenetic programming.

## 4. Materials and Methods

### 4.1. Animals and Tissue Collection

In vivo experiments were performed on a validated animal model of methyl donor deficiency [47,48]. They were conducted in compliance with the international guidelines for the care and use of laboratory animals and were approved by the local University Research Ethics Board. Wistar rats (Charles River, l′Arbresle, France) were maintained under standard laboratory conditions, on a 12-h light/dark cycle, with food and water available ad libitum. One month before mating, adult females were fed either a standard diet (Maintenance diet M20, Scientific Animal Food and Engineering, Villemoisson-sur-Orge, France) or a methyl donor deficient (MDD) low-choline diet (119 mg/kg vs. 1780 mg/kg) lacking folate and vitamin B12 (Special Diet Service, Saint-Gratien, France). Methionine content (~0.4%) was similar in both diets. The assigned diets were provided to the dams until weaning of the offspring at postnatal day 21. In the supplementation protocol, folic acid (the synthetic form of folate, Sigma-Aldrich, Saint-Quentin Fallavier, France) diluted in condensed milk was given per os at the dose of 3 mg/kg per day in a final volume of 1 mL to dams from embryonic days (E) 13 to postnatal day 21. Matched control dams received the same volume of vehicle (i.e., 1 mL condensed milk) over the same period. Whatever the maternal diet, pups were euthanized by excess isoflurane at 21 days of age and blood was withdrawn for subsequent plasma measurements. In some experiments, fetuses were collected at E20. Individuals were weighed and evaluated morphologically with the aid of a BX51WI microscope (Olympus, Rungis, France) coupled to a ProgRes MF cool camera (Jenoptik, Jena, Germany). For biochemical analyses, the brains were rapidly harvested and dissected before freezing in liquid nitrogen and stored at −80 °C. For immunochemistry, brains were immediately fixed in 4%-paraformaldehyde (24–48 h) at 4 °C, dehydrated and included in paraffin. Microtome-generated 12-μm sagittal brain sections were then mounted onto glass slides and stored at ambient temperature.

### 4.2. Measurement of Plasma Concentrations of Homocysteine, Vitamin B12, and Folate 

Homocysteine concentrations were measured by HPLC (Waters, St. Quentin, France) coupled with mass spectrometry (Api 4000 Qtrap; Applied Biosystems, Courtaboeuf, France) [49]. Vitamin B12 and folate concentrations were measured by radio-dilution isotope assay (simulTRAC-SNB; ICN Pharmaceuticals, Versailles, France) [50]. 

### 4.3. Histopathological Analyses

For basic histopathological investigations, brain sections were stained with the DNA fluorochrome 4,6-diamidino-2-phenylindole (Dapi, Sigma-Aldrich) for the measurement of thickness of specific brain layers.

For skeleton analysis, the procedure adapted from Wallin et al. [51] involves the complete skinning of fresh eviscerated rat embryos following a 24 h-immersion in 95% ethanol. Skeletons were stained for 48 h with 1% Alcian blue dye, diluted in an ethanol/acetic acid mix, specific for cartilage staining. Skeletons were then macerated in 2% KOH until bones are visible and stained for 24 h with 0.12% Alizarin red dye diluted in KOH, specific for bone staining. Specimen were finally cleared and hardened in glycerol/ethanol baths and stored in 87% glycerol. Detailed observations and measurements were performed by means of the Cell^®^ software (Olympus).

### 4.4. RNA Extraction

Total RNA was extracted from 0.5 mg of hippocampus using the mirVana^®^miRNA Isolation kit (Applied Biosystems, Foster City, CA, USA) following the manufacturer′s instructions, and as previously described by Kerek et al. [22]. miRNAs were isolated using a two-step procedure. In the first step, samples were disrupted in a denaturing lysis buffer, and then subjected to acid-phenol/chloroform extraction. The second step consisted of purification over glass-fiber filter that immobilizes the RNA which was later eluted using RNase-free water. According to the manufacturer’s instructions, no enrichment procedure is needed while isolating miRNA for expression profiling using miRNA arrays. The concentration and purity of RNA were determined at 260/280 nm by using a nanodrop spectrophotometer (Multiskan GO, Thermo Fisher, Illkirch, France).

### 4.5. Analysis of let-7a, miR-34a and miR-23a Expression

TaqMan RT-qPCR: Two-step real-time PCR was used to analyze the expression of microRNAs. In the first step total RNA was reverse transcribed using miRNA-specific RT primers (rno-miR-34, rno-miR-23 and U6SnoRNA) and a TaqMan^®^ MicroRNA Reverse Transcription Kit (Applied Biosystems, Villebon-sur-Yvette, France). miRNA expression was analyzed using TaqMan microRNA assays (Applied Biosystems), according to the instructions of the manufacturer. The RT reaction was performed in 15 µL volume, containing 1µg RNA sample, 3 µL primer, and master mix adjusted to 15 µL/reaction. Products of RT reaction (1.33 µL) were used in a real-time PCR reaction, which also included 10 µL of the TaqMan Universal Master Mix II, and 1 µL TaqMan miRNA assay containing the sequence-specific primers of either the target miRNA (let-7a: UGAGGUAGUAGGUUGUAUAGUU, miR-34a: UGGCAGUGUCUUAGCUGGUUGU, and miR-23a: AUCACAUUGCCAGGGAUUUCC) or the U6SnoRNA (CACGAATTTGCGTGTCATCCTT) used as an endogenous control for normalization. Real-time PCR was carried out by means of a Step One Plus Real-Time PCR System (Applied Biosystems). Incubations were performed in a 96-well plate at 95 °C for 10 min for enzyme activation, followed by 40 cycles of PCR: Denaturation (95 °C for 15 s), and annealing/extending (60 °C for 2 min). Data analysis was performed with the software provided by the manufacturer (Step One Plus).

In situ hybridization: The in situ detection of let-7a, miR-34a and miR-23a was performed on paraffin embedded sections from normal and methyl donor deficient brain tissues by locked nucleic acid (LNA)-oligo in situ hybridization, as previously described by Kloosterman et al. [52]. Briefly, the slides were deparaffinized in xylene, rehydrated in decreasing concentrations of ethanol, and treated with proteinase-k for nucleic acid release. Slides were then re-dehydrated and prehybridized in hybridization buffer with 0.5 nm specific probe (LNA-modified and digoxygenin (DIG)-labeled oligonucleotide, Exiqon, Copenhagen, Denmark) complementary to let-7a (AACTATACAACCTACTACCTCA), miR-34a (ACAACCAGCTAAGACACTGCCA or miR-23a (GGAAATCCCTGGCAATGTGAT). Sections were then washed in saline sodium citrate buffer, followed by blocking in Denhardt solution 1× in a humidified chamber. Slides were then incubated with anti-DIG antibody (1/500, Roche Applied Science, Basel, Switzerland) for 1 h at room temperature, washed in PBS-T and then immunoreactivity was assessed in the presence of a matching secondary antibody conjugated to AlexaFluor (1/2000, Molecular Probes, Eugene, OR, USA) for 1 h at room temperature. Positive controls (snoRNA U6B, Exiqon: CACGAATTTGCGTGTCATCCTT) were used for each hybridization experiment.

### 4.6. Behavioral Evaluation 

The static righting reflex was studied as described by Blaise et al. [47]. The time needed by the pup to right itself in a supine position was recorded at postnatal days 5 and 7.

The negative geotaxis was tested from postnatal day 7 to postnatal day 11. The rat pup was positioned with the head downward on an inclined plane with a 20% slope. The time needed for the pup to turn completely and reach a position with the head upward on the plane was measured. The duration of the test was limited to 120 s.

The learning performances were evaluated using a water maze. The apparatus consists of a square pool filled with water (5 cm maintained at 25 °C). Grey plastic walls 30 cm high were used to delimit 25 square zones equal in size (15 × 15 cm). Open doors in walls allowed communications between zones designing an ideal route from a starting zone to an exit one with additional lateral error-zones. Each pup was allowed to run the maze twice a day during 4 consecutive days from day 21 to day 24 (sessions S1 to S4 with a cut-off time of 2 min). Parameters recorded were the time used to run the maze from the starting zone to the exit one, named “escape latency” (if no exit was recorded, the cut-off time was attributed); the number of errors committed (one or more entries in zones outside of the ideal route). Two days before session one, each pup was allowed to know the apparatus in two habituation sessions (not recorded).

### 4.7. Statistical Analysis

Data were analyzed with Statview 5 software for Windows (SAS Institute, Berkley, CA, USA). They were compared by using one-way analysis of variance (ANOVA) with Fisher’s test. *p*-value < 0.05 was considered to indicate significance.

## 5. Conclusions

Whereas folate supplementation during the last third of gestation in rats was demonstrated to alleviate methyl donor deficiency-related birth defects, our present study suggests that its continuation after birth may help to reduce growth retardation and to ameliorate neurological symptoms commonly associated with developmental deficiencies in folate and vitamin B12.
